# The AGL6–ELF3–FT circuit controls flowering time in *Arabidopsis*

**DOI:** 10.1080/15592324.2024.2358684

**Published:** 2024-05-28

**Authors:** Kyounghee Lee, Hobin Yoon, Pil Joon Seo

**Affiliations:** a Department of Chemistry, Seoul National University, Seoul, Republic of Korea; b Research Institute of Basic Sciences, Seoul National University, Seoul, Republic of Korea; c Plant Genomics and Breeding Institute, Seoul National University, Seoul, Republic of Korea

**Keywords:** *Arabidopsis*, flowering, AGL6, ELF3, FT

## Abstract

Adjusting the timing of floral transition is essential for reproductive success in plants. A number of flowering regulators integrate internal and external signals to precisely determine the time to flower. We here report that the AGAMOUS-LIKE 6 (AGL6) – EARLY FLOWERING 3 (ELF3) module regulates flowering in the FLOWERING LOCUS T (FT)-dependent pathway in *Arabidopsis*. The AGL6 transcriptional repressor promotes floral transition by directly suppressing *ELF3*, which in turn directly represses *FT* expression that acts as a floral integrator. Indeed, *ELF3* is epistatic to *AGL6* in the control of floral transition. Overall, our findings propose that the AGL6–ELF3 module contributes to fine-tuning flowering time in plants.

Coordinating the timing of the transition from vegetative to reproductive stages is essential for plants to reproduce offspring. Internal and external cues mediated by intricate signaling networks are integrated to the *FT* gene encoding a phosphatidylethanolamine-binding protein that acts as a key floral integrator, precisely determining flowering time.^1^ Under inductive conditions, *FT* is expressed in leaf vasculature and systemically transported through phloem to shoot apical meristem (SAM).^2–5^ In SAM, the FT protein interacts with the SAM-specific bZIP transcription factor FD to activate downstream genes, such as *APETALA 1* (*AP1*) and *LEAFY* (*LFY*), allowing the transition from vegetative SAM to inflorescence meristem.^1,6–8^

CO and FLC are the central floral regulators that antagonistically regulate *FT* expression.^9^ The floral activator CO, a nuclear protein containing a CCT motif and two B-box-type zinc-finger domains, directly binds to the *FT* locus and activates its expression.^10^ *CO* expression shows a peak during late afternoon under both long-day (LD) and short-day (SD) conditions. However, accumulation of CO protein is dependent on light exposure, and thus its function is enhanced only under LD conditions, facilitating photoperiodic flowering control.^11–13^ In parallel, a MADS-box transcription factor, FLC is a representative repressor of floral transition, which directly suppresses *FT* expression. *FLC* sub-integrates internal and external signals mediated by autonomous and vernalization pathways.^14–16^

The AGL6 MADS-domain transcription factor is known as a floral activator, which is likely conserved across many plant species.^17–19^ Ectopic expression of *AGL6* results in early flowering with upregulation of *FT* expression. The early flowering phenotype of *agl6-1D* is completely suppressed by introducing *ft* mutations, supporting that *FT* is epistatic to *AGL6* .^17^ However, several genetic studies have demonstrated that *AGL6* acts additively to *CO* and *FLC* in the control of floral transition, suggesting that AGL6 may activate *FT* expression in addition to FLC- and CO-dependent pathways during floral transition.^17^ Thus, we wanted to know a signaling pathway accounting for AGL6-regulated flowering time.

Given that 35S:*AGL6-EAR* transgenic plants, in which the *AGL6* coding sequence fused to the EAR transcriptional repressor motif is ectopically expressed, phenotypically mimic *AGL6*-overexpressing transgenic plants that display early flowering,^18^ AGL6 acts as a transcriptional repressor and most likely inhibits the expression of a floral suppressor. To find out a regulatory target of AGL6, we searched for all known floral suppressors; among others, we were interested in *EARLY FLOWERING 3* (*ELF3*), since the ELF3 protein is known to repress floral transition by repressing *FT* expression independently of *CO*.^20,21^ To check whether *ELF3* is regulated by AGL6, we first examined *ELF3* expression in 35S:*AGL6* transgenic plants. As a result, *ELF3* expression was indeed repressed in 35S:*AGL6* transgenic plants compared with wild type (Figure 1(a), Supplementary Figure S1), while *AGL6* expression was unaffected in 35S:*ELF3-YFP* transgenic plants (Figure 1(b)), suggesting that *AGL6* acts upstream of *ELF3*.

**Figure 1.**
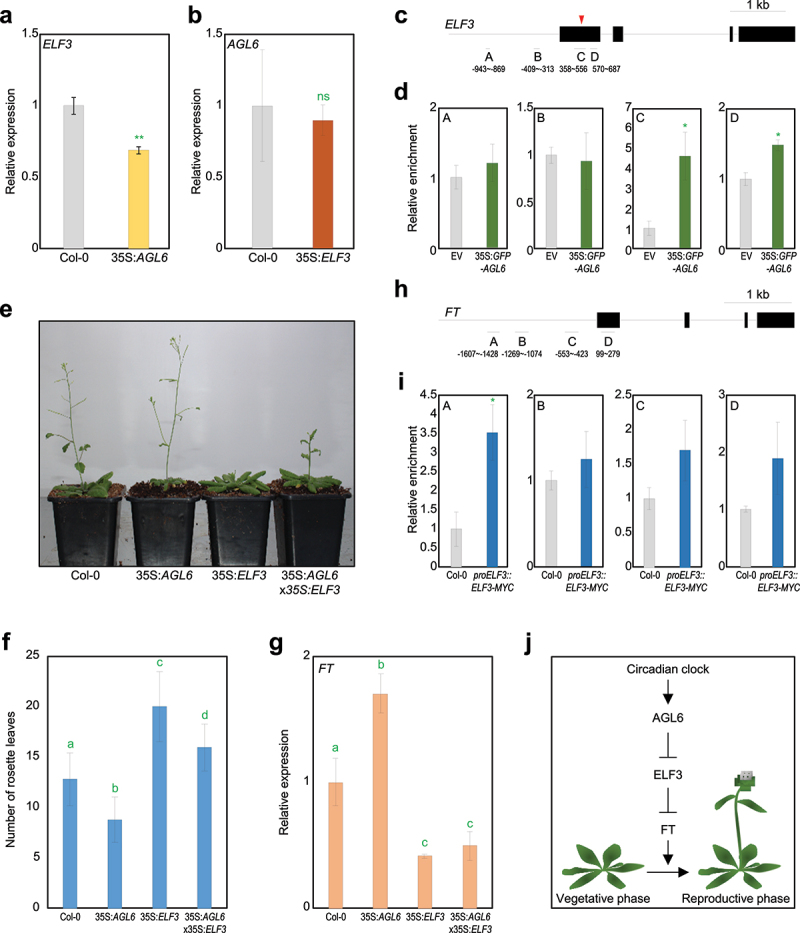
AGL6 directly represses *ELF3* in the control of floral transition. A *ELF3* expression in wild-type and 35S:*AGL6* plants. b *AGL6* expression in wild-type and 35S:*ELF3-YFP* plants. In a and b, eight-day-old seedlings grown under long-day (LD) conditions were harvested at ZT13 for total RNA isolation. Transcript accumulation was analyzed by RT-qPCR. The *eIF4a* gene was used as an internal control. Data indicate mean ± SEM. Asterisks indicate statistically significant differences (**p < .01; ns, not significant; Student’s *t*-test). c Structure of *ELF3* gene. Black lines above the labels indicate regions amplified by quantitative PCR (qPCR) following chromatin immunoprecipitation (ChIP). Black boxes indicate exons. Red arrowhead represents degenerated CArG-box motif. d Enrichment of AGL6 in *ELF3* locus. Arabidopsis protoplasts isolated from 2-week-old seedlings were transfected with 35S:*GFP-AGL6* construct and empty vector control. Values obtained from control plants were set to 1 after normalization against *eIF4a*. Data indicate mean ± SEM. Asterisks indicate statistically significant differences (*p < .05; Student’s *t*-test). e Flowering phenotype of wild-type, 35S:*AGL6*, 35S:*ELF3-YFP* (35S:*ELF3*), and 35S:*AGL6 ×* 35S:*ELF3-YFP* under LD condition. f Measurement of rosette leaf numbers. Flowering time was measured by counting the total number of rosette leaves at flowering initiation. Data indicate mean ± SEM. Statistically significant differences were determined using one-way analysis of variance (ANOVA), followed by Newman–Keuls’s *post hoc* test. Different letters indicate significant differences (*p < .05). g *FT* expression in wild-type, 35S:*AGL6*, 35S:*ELF3-YFP* (35S:*ELF3*), and 35S:*AGL6 ×* 35S:*ELF3-YFP*. Eight-day-old seedlings grown under long day condition were harvested at ZT9 for total RNA isolation. Data indicate mean ± SEM. Statistically significant differences were determined using one-way analysis of variance (ANOVA), followed by Newman–Keuls’s *post hoc* test. h Structure of *FT* gene. i Enrichment of ELF3 in *FT* locus. Data indicate mean ± SEM. Asterisks indicate statistically significant differences (*p < .05; Student’s *t*-test). j Proposed model showing regulation of floral transition by AGL6. AGL6 directly represses *ELF3* expression, which in turn directly represses *FT* expression. The AGL6-ELF3 module is likely related to circadian-regulated flowering responses.

To examine whether AGL6 directly binds to the *ELF3* locus, we performed chromatin immunoprecipitation-quantitative PCR (ChIP-qPCR) assays using *Arabidopsis* protoplasts transiently expressing the 35S:*GFP-AGL6* construct. As a result, AGL6 associated around transcription start site of the *ELF3* locus (Figure 1(c,d)), where a MADS domain-binding CArG-box motif exists.^22^ These results indicate that AGL6 directly binds to the *ELF3* gene to repress its expression.

To prove the genetic hierarchy between *AGL6* and *ELF3* in the control of floral transition, we genetically crossed 35S:*AGL6* transgenic plants with 35S:*ELF3-YFP* transgenic plants and measured total rosette leaf numbers. Under LD conditions, the floral transition of 35S:*AGL6* transgenic plants was accelerated, whereas 35S:*ELF3-YFP* transgenic plants exhibited delayed floral transition compared with wild type (Figure 1(e,f)). Notably, early flowering phenotype of 35S:*AGL6* transgenic plants was impaired in 35S:*AGL6* x 35S:*ELF3-YFP* (Figure 1(e,f)). Increased *FT* expression in 35S:*AGL6* transgenic plants was also compromised in 35S:*AGL6 ×* 35S:*ELF3-YFP* plants, which was equivalent to that of 35S:*ELF3-YFP* transgenic plants (Figure 1(g)).

Since ELF3 strongly represses floral transition independently of *CO*,^21^ we were curious whether ELF3 directly represses *FT* expression. ChIP-qPCR assays using *proELF3:ELF3-MYC* transgenic plants revealed that ELF3 bound directly to the *FT* promoter (Figure 1(h,i)), indicating that ELF3 represses *FT* expression through a direct binding to its promoter. Overall, our study demonstrates that AGL6 activates indirectly *FT* expression, at least in part through direct association with *ELF3* to promote floral transition (Figure 1(j)).

Although both *AGL6-ox* transgenic and *elf3* mutant plants display photoperiod-insensitive flowering,^17,20^ transcript and protein accumulation of AGL6 was unchanged by day length (Supplementary Figure S2 and S3).^17^ Alternatively, the AGL6-ELF3 module likely plays a role in fine-tuning circadian clock-mediated photoperiodic flowering in plants. The night-expressed *ELF3* gene is well-known to control circadian oscillation,^23^ and *AGL6* also showed diurnal expression with a peak at 8 h after dawn (Supplementary Figure S4). Their complementary expression patterns might be related to the repression of *ELF3* by AGL6. Although it is currently elusive what input signals regulate AGL6 activity, the AGL6-ELF3-FT circuit may constitute a diurnal flowering response pathway. In addition, this study also needs to be substantiated with future research, including functional analysis of *agl6* single or high-order mutants as well as detailed analysis of functional and genetic relationships between *AGL6* and *ELF3*.

## Materials and methods

### Plant material and growth conditions

*Arabidopsis thaliana* (Columbia-0 ecotype for all experiments unless otherwise specified) seeds were obtained from *Arabidopsis* Biological Resource Center (ABRC; https://abrc.osu.edu/), which distributes diverse seed stocks of Arabidopsis thaliana and related species. Plants were grown under long-day (LD) condition (16-h light/8-h dark cycles) and with cool white fluorescent light (100 μmol photons m^−2^ s^−1^) at 23°C. The *proELF3:ELF3-MYC/elf3–1* plants have been described previously.^24^ The 35S:*AGL6* transgenic plants were generated by *Agrobacterium*-mediated transformation.

To analyze the floral transition, plants were grown on soil under LD conditions and counted the total number of rosette leaves at floral transition. At least 30 plants were measured and averaged for each plant genotype.

Experimental research on plants including the collection of plant material was performed in accordance with relevant institutional, national, and international guidelines and legislation.

## Quantitative real-time RT-PCR analysis

Total RNA was extracted using TransZol Up (Transgen Biotech) according to the manufacturer’s recommendations. After treatment of total RNAs with RNAse-free DNAse (NEB), reverse transcription (RT) was performed using Moloney Murine Leukemia Virus (M-MLV) reverse transcriptase (Enzynomics) with oligo(dT18). Quantitative RT-PCR (RT-qPCR) reactions were performed in 96-well blocks using the Step-One Plus Real-Time PCR System (Applied Biosystems). The qPCR primers used are listed in Supplementary Table S1. The values for each set of primers were normalized relative to the *EUKARYOTIC TRANSLATION INITIATION FACTOR 4A1* (*eIF4A*) gene (At3g13920).

## Protoplast isolation

The protoplast isolation was performed as previously reported with some modifications.^25^ Two-week-old seedlings grown under LD conditions were digested in 20 mL enzyme solution (2% Viscozyme L, 1% Celluclast 1.5 L, 1% Pectinex Ultra SP-L in MMC, adjusted to pH 5.8 by NaOH and sterilized through a 0.2 μm syringe filtering) and incubated at room temperature for 5 h with gentle shaking to isolate mesophyll protoplasts. The protoplasts were collected by centrifugation at 100 g for 7 min and washed twice with the W5 solution containing 0.1% glucose, 0.08% KCl, 0.9% NaCl, 1.84% CaCl_2_, and 2 mM MES (pH 5.7). The 35S:*GFP-AGL6* construct was transiently transfected into protoplasts through the PEG-mediated transfection method. After 16-h incubation in the dark at 23°C for the AGL6 protein expression, protoplasts were harvested for ChIP assays.

## Chromatin immunoprecipitation (ChIP) assays

Chromatin immunoprecipitation (ChIP) assays were performed as previously described.^26^ To immunoprecipitate AGL6 protein in protoplasts transfected with 35S:*GFP-AGL6* construct, we used the salmon sperm DNA/protein A agarose beads (Millipore) and anti-GFP (Abcam). For ChIP using *proELF3:ELF3-MYC/elf3–1* transgenic plants, ELF3 protein was immunoprecipitated using magnetic beads coated with an anti-MYC-tag antibody (88842; invitrogen). DNA was purified using DNA purification kit (Cosmogenetech). The level of precipitated DNA fragments was quantified by qPCR using specific primer sets (Supplementary Table S2). The values were normalized as relative comparisons to *eIF4a* values.

## Immunoblot analysis

Total proteins were extracted from Arabidopsis mesophyll protoplasts transfected with 35S:*GFP-AGL6* construct using SDS – PAGE loading buffer. The protein samples were then loaded onto 10% polyacrylamide gel and separated by SDS – PAGE, as previously described.^26^ Protein samples in the gel were transferred onto Hybond-P+ membranes (Amersham Pharmacia). Epitope-tagged proteins were immunologically detected using an anti-GFP antibody (ab290; Abcam).

## Statistical analysis

Statistical analysis of all data in the study was conducted through GraphPad Prism 8 software.

## Supplementary Material

Supplemental_Figures.pptx

## Data Availability

All relevant data can be found within the manuscript and its supporting materials.

## References

[1] Gonzalez-Suarez P, Walker CH, Bennett T. FLOWERING LOCUS T mediates photo-thermal timing of inflorescence meristem arrest in Arabidopsis thaliana. Plant Physiol. 2023;192(3):2276–5. doi:10.1093/plphys/kiad163. 36943252 PMC10315265

[2] Corbesier L, Vincent C, Jang S, Fornara F, Fan Q, Searle I, Giakountis A, Farrona S, Gissot L, Turnbull C. et al. FT protein movement contributes to long-distance signaling in floral induction of Arabidopsis. Science. 2007;316(5827):1030–1033. doi:10.1126/science.1141752. 17446353

[3] Jaeger KE, Wigge PA. FT protein acts as a long-range signal in Arabidopsis. Curr Biol. 2007;17(12):1050–1054. doi:10.1016/j.cub.2007.05.008. 17540569

[4] Mathieu J, Warthmann N, Kuttner F, Schmid M. Export of FT protein from phloem companion cells is sufficient for floral induction in Arabidopsis. Curr Biol. 2007;17(12):1055–1060. doi:10.1016/j.cub.2007.05.009. 17540570

[5] Giakountis A, Coupland G. Phloem transport of flowering signals. Curr Opin Plant Biol. 2008;11(6):687–694. doi:10.1016/j.pbi.2008.10.003. 18977685

[6] Wigge PA, Kim MC, Jaeger KE, Busch W, Schmid M, Lohmann JU, Weigel D. Integration of spatial and temporal information during floral induction in Arabidopsis. Science. 2005;309(5737):1056–1059. doi:10.1126/science.1114358. 16099980

[7] Andres F, Romera-Branchat M, Martínez-Gallegos R, Patel V, Schneeberger K, Jang S, Altmüller J, Nürnberg P, Coupland G. Floral induction in Arabidopsis by FLOWERING LOCUS T requires direct repression of BLADE-ON-PETIOLE genes by the homeodomain protein PENNYWISE. Plant Physiol. 2015;169:2187–2199. doi:10.1104/pp.15.00960. 26417007 PMC4634070

[8] Takagi H, Hempton AK, Imaizumi T. Photoperiodic flowering in Arabidopsis: multilayered regulatory mechanisms of CONSTANS and the florigen FLOWERING LOCUS T. Plant Commun. 2023;4(3):100552. doi:10.1016/j.xplc.2023.100552. 36681863 PMC10203454

[9] Lee J, Lee I. Regulation and function of SOC1, a flowering pathway integrator. J Exp Bot. 2010;61(9):2247–2254. doi:10.1093/jxb/erq098. 20413527

[10] Yoo SK, Chung KS, Kim J, Lee JH, Hong SM, Yoo SJ, Yoo SY, Lee JS, Ahn JH. Constans activates suppressor of overexpression of constans 1 through Flowering Locus T to promote flowering in Arabidopsis. Plant Physiol. 2005;139(2):770–778. doi:10.1104/pp.105.066928. 16183837 PMC1255994

[11] Jang S, Marchal V, Panigrahi KCS, Wenkel S, Soppe W, Deng X-W, Valverde F, Coupland G. Arabidopsis COP1 shapes the temporal pattern of CO accumulation conferring a photoperiodic flowering response. Embo J. 2008;27(8):1277–1288. doi:10.1038/emboj.2008.68. 18388858 PMC2291449

[12] Sawa M, Kay SA, Imaizumi T. Photoperiodic flowering occurs under internal and external coincidence. Plant Signal Behav. 2008;3(4):269–271. doi:10.4161/psb.3.4.5219. 19704651 PMC2634199

[13] Song YH, Estrada DA, Johnson RS, Kim SK, Lee SY, MacCoss MJ, Imaizumi T. Distinct roles of FKF1, Gigantea, and Zeitlupe proteins in the regulation of Constans stability in Arabidopsis photoperiodic flowering. Proc Natl Acad Sci USA. 2014;111(49):17672–17677. doi:10.1073/pnas.1415375111. 25422419 PMC4267339

[14] Searle I, He Y, Turck F, Vincent C, Fornara F, Kröber S, Amasino RA, Coupland G. The transcription factor FLC confers a flowering response to vernalization by repressing meristem competence and systemic signaling in Arabidopsis. Genes Dev. 2006;20(7):898–912. doi:10.1101/gad.373506. 16600915 PMC1472290

[15] Wu Z, Fang X, Zhu D, Dean C. Autonomous pathway: FLOWERING LOCUS C repression through an antisense-mediated chromatin-silencing mechanism. Plant Physiol. 2020;182(1):27–37. doi:10.1104/pp.19.01009. 31740502 PMC6945862

[16] Ratcliffe OJ, Nadzan GC, Reuber TL, Riechmann JL. Regulation of flowering in Arabidopsis by an FLC homologue. Plant Physiol. 2001;126(1):122–132. doi:10.1104/pp.126.1.122. 11351076 PMC102287

[17] Yoo SK, Wu X, Lee JS, Ahn JH. AGAMOUS-LIKE 6 is a floral promoter that negatively regulates the FLC/MAF clade genes and positively regulates FT in Arabidopsis. Plant J. 2011;65(1):62–76. doi:10.1111/j.1365-313X.2010.04402.x. 21175890

[18] Koo SC, Bracko O, Park MS, Schwab R, Chun HJ, Park KM, Seo JS, Grbic V, Balasubramanian S, Schmid M. et al. Control of lateral organ development and flowering time by the Arabidopsis thaliana MADS-box gene AGAMOUS-LIKE6. Plant Journal. 2010;62(5):807–816. doi:10.1111/j.1365-313X.2010.04192.x. 20230491

[19] Wang L, Song J, Han X, Yu Y, Wu Q, Qi S, Xu Z. Functional divergence analysis of AGL6 genes in Prunus mume. Plants (Basel). 2022;12(1):158. doi:10.3390/plants12010158. 36616287 PMC9824310

[20] Yu JW, Rubio V, Lee N-Y, Bai S, Lee S-Y, Kim S-S, Liu L, Zhang Y, Irigoyen ML, Sullivan JA. et al. COP1 and ELF3 control circadian function and photoperiodic flowering by regulating GI stability. Mol Cell. 2008;32(5):617–630. doi:10.1016/j.molcel.2008.09.026. 19061637 PMC2651194

[21] Kim WY, Hicks KA, Somers DE. Independent roles for EARLY FLOWERING 3 and ZEITLUPE in the control of circadian timing, hypocotyl length, and flowering time. Plant Physiol. 2005;139(3):1557–1569. doi:10.1104/pp.105.067173. 16258016 PMC1283789

[22] Batista RA, Moreno-Romero J, Qiu Y, van Boven J, Santos-González J, Figueiredo DD, Köhler C. The MADS-box transcription factor PHERES1 controls imprinting in the endosperm by binding to domesticated transposons. Elife. 2019;8. doi:10.7554/eLife.50541. PMC6914339 31789592

[23] Nusinow DA, Helfer A, Hamilton EE, King JJ, Imaizumi T, Schultz TF, Farré EM, Kay SA. The ELF4–ELF3–LUX complex links the circadian clock to diurnal control of hypocotyl growth. Nature. 2011;475(7356):398–402. doi:10.1038/nature10182. 21753751 PMC3155984

[24] Tong M, Lee K, Ezer D, Cortijo S, Jung J, Charoensawan V, Box MS, Jaeger KE, Takahashi N, Mas P. et al. The evening complex establishes repressive chromatin domains via H2A.Z deposition. Plant Physiol. 2020;182(1):612–625. doi:10.1104/pp.19.00881. 31712305 PMC6945876

[25] Jeong YY, Lee HY, Kim SW, Noh YS, Seo PJ. Optimization of protoplast regeneration in the model plant Arabidopsis thaliana. Plant Methods. 2021;17(1):21. doi:10.1186/s13007-021-00720-x. 33622383 PMC7901198

[26] Lee K, Park O-S, Go JY, Yu J, Han JH, Kim J, Bae S, Jung YJ, Seo PJ. Arabidopsis ATXR2 represses de novo shoot organogenesis in the transition from callus to shoot formation. Cell Rep. 2021;37(6):109980. doi:10.1016/j.celrep.2021.109980. 34758306

