# Supplementary material for: The AGL6–ELF3–FT circuit controls flowering time in Arabidopsis
Source: Plant Signal Behav. 2024 May 28;19(1):2358684. doi: 10.1080/15592324.2024.2358684 (PMC11135843; doi:10.1080/15592324.2024.2358684)
Supplement: Supplemental_Figures.pptx [file KPSB_A_2358684_SM5614.pptx]

## Slide 1
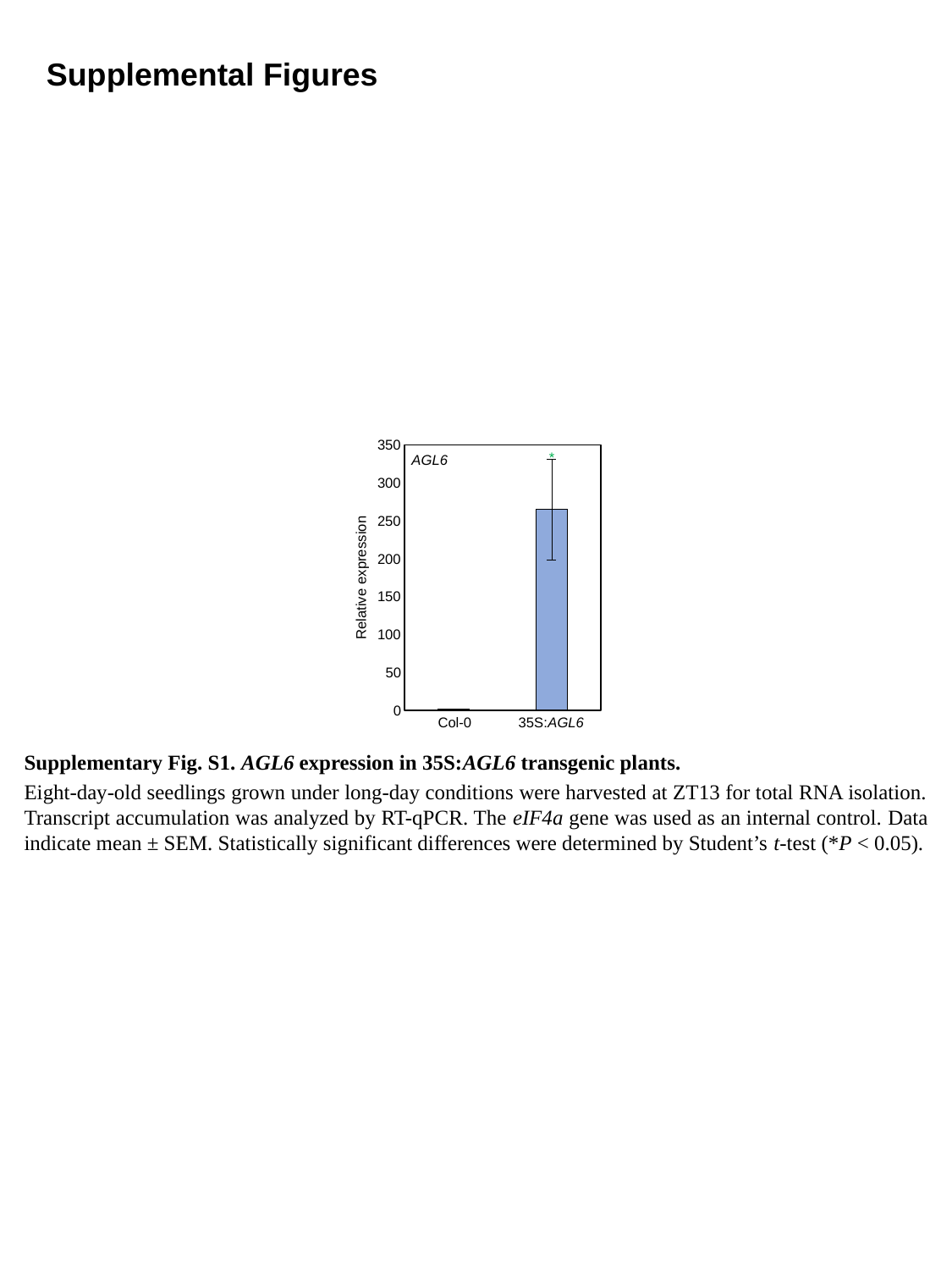

Supplemental Figures
350
*
AGL6
300
250
200
Relative expression
150
100
50
0
Col-0
35S:AGL6
Supplementary Fig. S1. AGL6 expression in 35S:AGL6 transgenic plants.
Eight-day-old seedlings grown under long-day conditions were harvested at ZT13 for total RNA isolation. Transcript accumulation was analyzed by RT-qPCR. The eIF4a gene was used as an internal control. Data indicate mean ± SEM. Statistically significant differences were determined by Student’s t-test (*P < 0.05).

## Slide 2
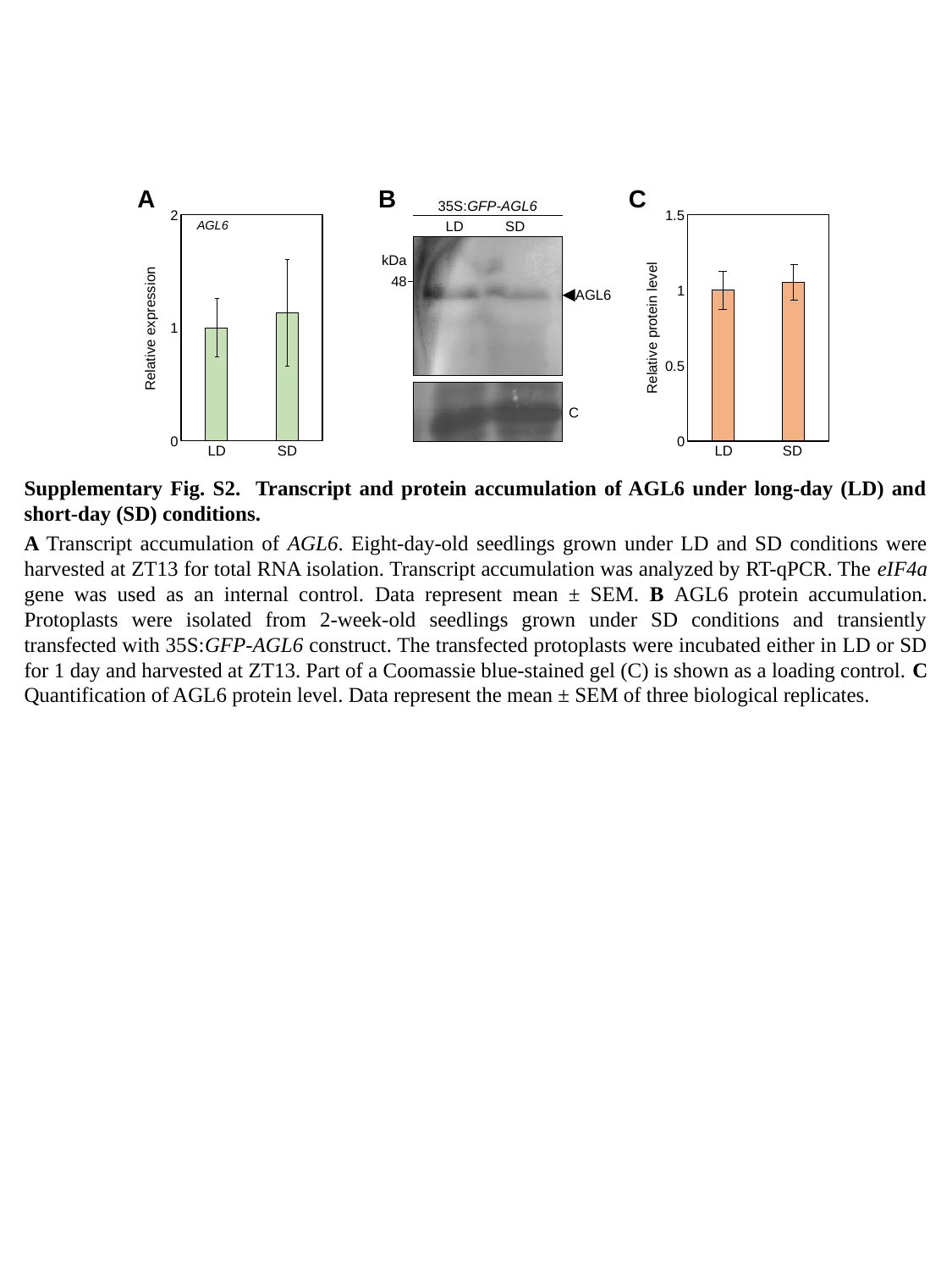

A
B
C
35S:GFP-AGL6
2
1.5
AGL6
LD
SD
kDa
48
1
AGL6
Relative protein level
1
Relative expression
0.5
C
0
0
LD
SD
LD
SD
Supplementary Fig. S2. Transcript and protein accumulation of AGL6 under long-day (LD) and short-day (SD) conditions.
A Transcript accumulation of AGL6. Eight-day-old seedlings grown under LD and SD conditions were harvested at ZT13 for total RNA isolation. Transcript accumulation was analyzed by RT-qPCR. The eIF4a gene was used as an internal control. Data represent mean ± SEM. B AGL6 protein accumulation. Protoplasts were isolated from 2-week-old seedlings grown under SD conditions and transiently transfected with 35S:GFP-AGL6 construct. The transfected protoplasts were incubated either in LD or SD for 1 day and harvested at ZT13. Part of a Coomassie blue-stained gel (C) is shown as a loading control. C Quantification of AGL6 protein level. Data represent the mean ± SEM of three biological replicates.

## Slide 3
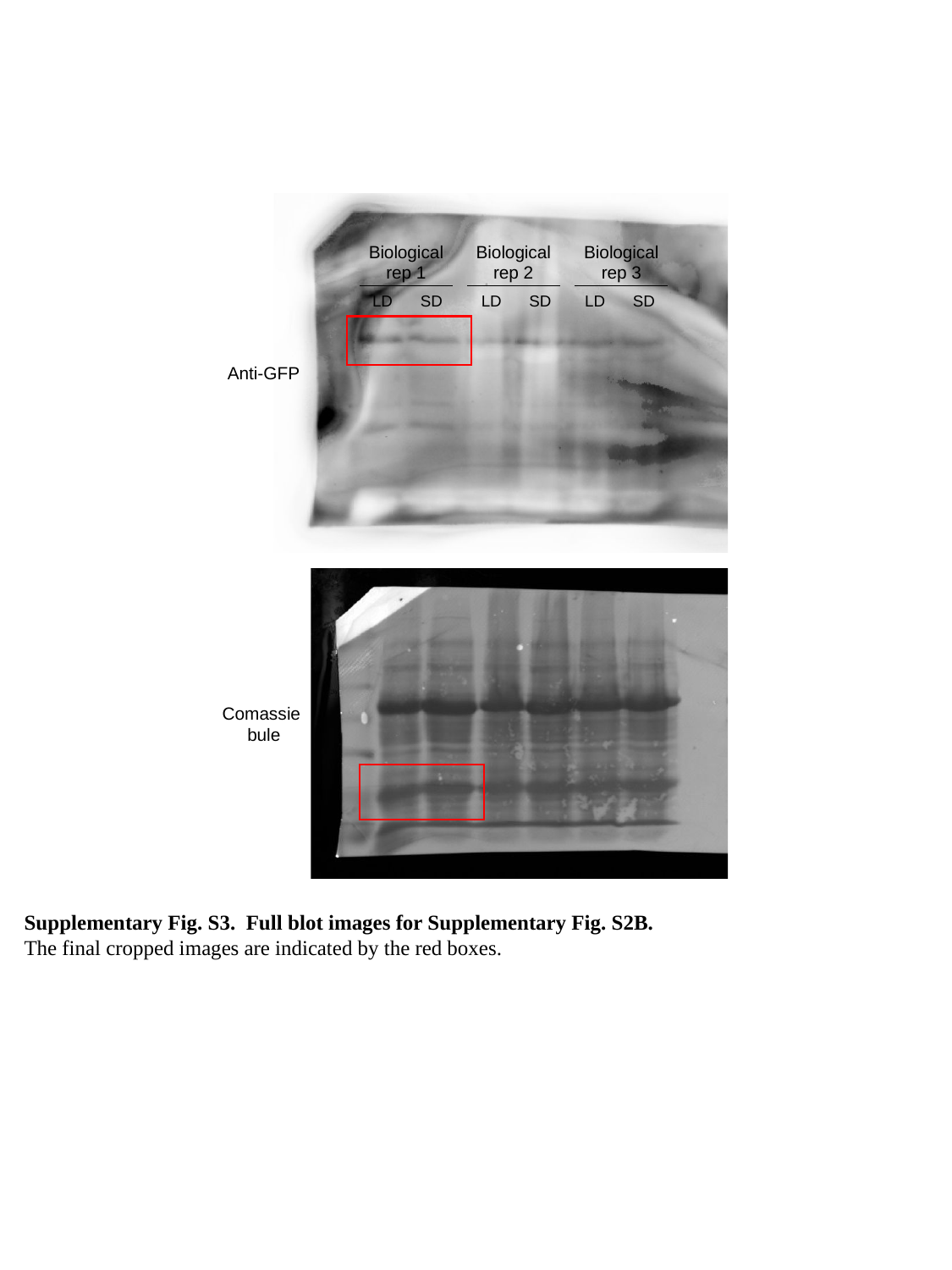

Biological
rep 1
Biological
rep 2
Biological
rep 3
LD
SD
LD
SD
LD
SD
Anti-GFP
Comassie
bule
Supplementary Fig. S3. Full blot images for Supplementary Fig. S2B.
The final cropped images are indicated by the red boxes.

## Slide 4
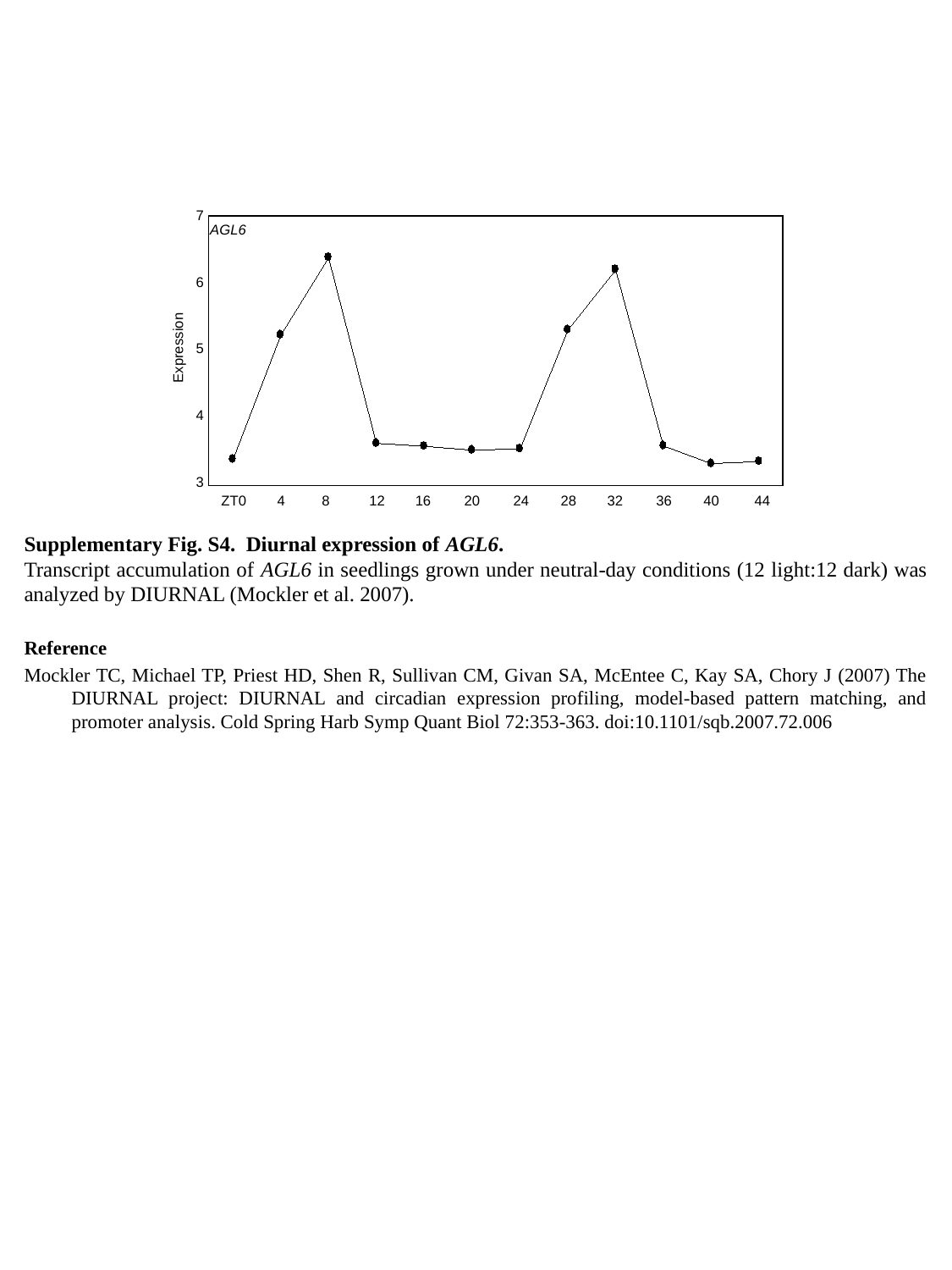

7
AGL6
6
Expression
5
4
3
ZT0
4
8
12
16
20
24
28
32
36
40
44
Supplementary Fig. S4. Diurnal expression of AGL6.
Transcript accumulation of AGL6 in seedlings grown under neutral-day conditions (12 light:12 dark) was analyzed by DIURNAL (Mockler et al. 2007).
Reference
Mockler TC, Michael TP, Priest HD, Shen R, Sullivan CM, Givan SA, McEntee C, Kay SA, Chory J (2007) The DIURNAL project: DIURNAL and circadian expression profiling, model-based pattern matching, and promoter analysis. Cold Spring Harb Symp Quant Biol 72:353-363. doi:10.1101/sqb.2007.72.006

## Slide 5
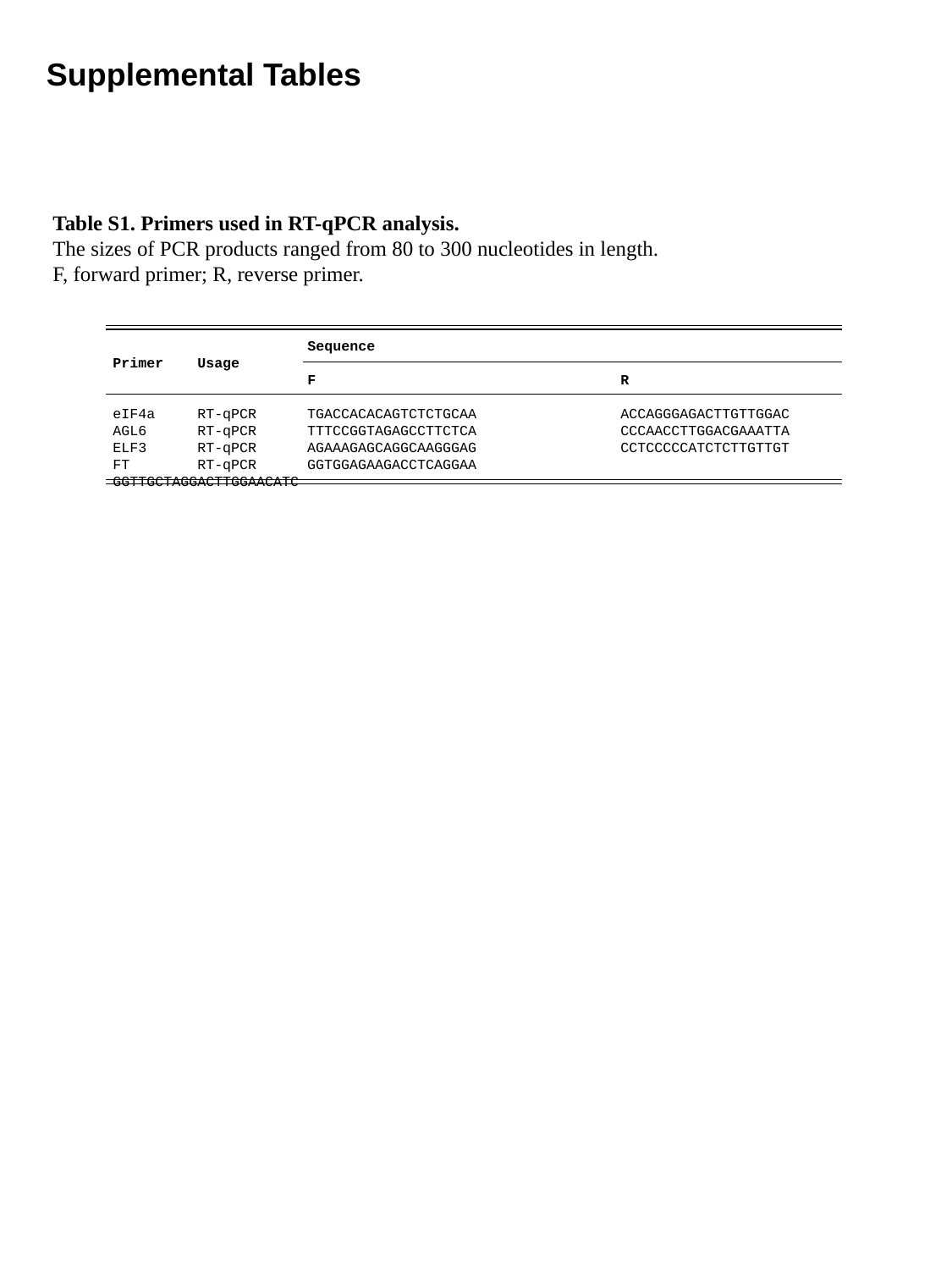

Supplemental Tables
Table S1. Primers used in RT-qPCR analysis.
The sizes of PCR products ranged from 80 to 300 nucleotides in length.
F, forward primer; R, reverse primer.
	 Sequence
Primer Usage
	 F	 	R
eIF4a RT-qPCR TGACCACACAGTCTCTGCAA 	ACCAGGGAGACTTGTTGGAC
AGL6 RT-qPCR TTTCCGGTAGAGCCTTCTCA		CCCAACCTTGGACGAAATTA
ELF3 RT-qPCR AGAAAGAGCAGGCAAGGGAG	 	CCTCCCCCATCTCTTGTTGT
FT RT-qPCR GGTGGAGAAGACCTCAGGAA 		GGTTGCTAGGACTTGGAACATC

## Slide 6
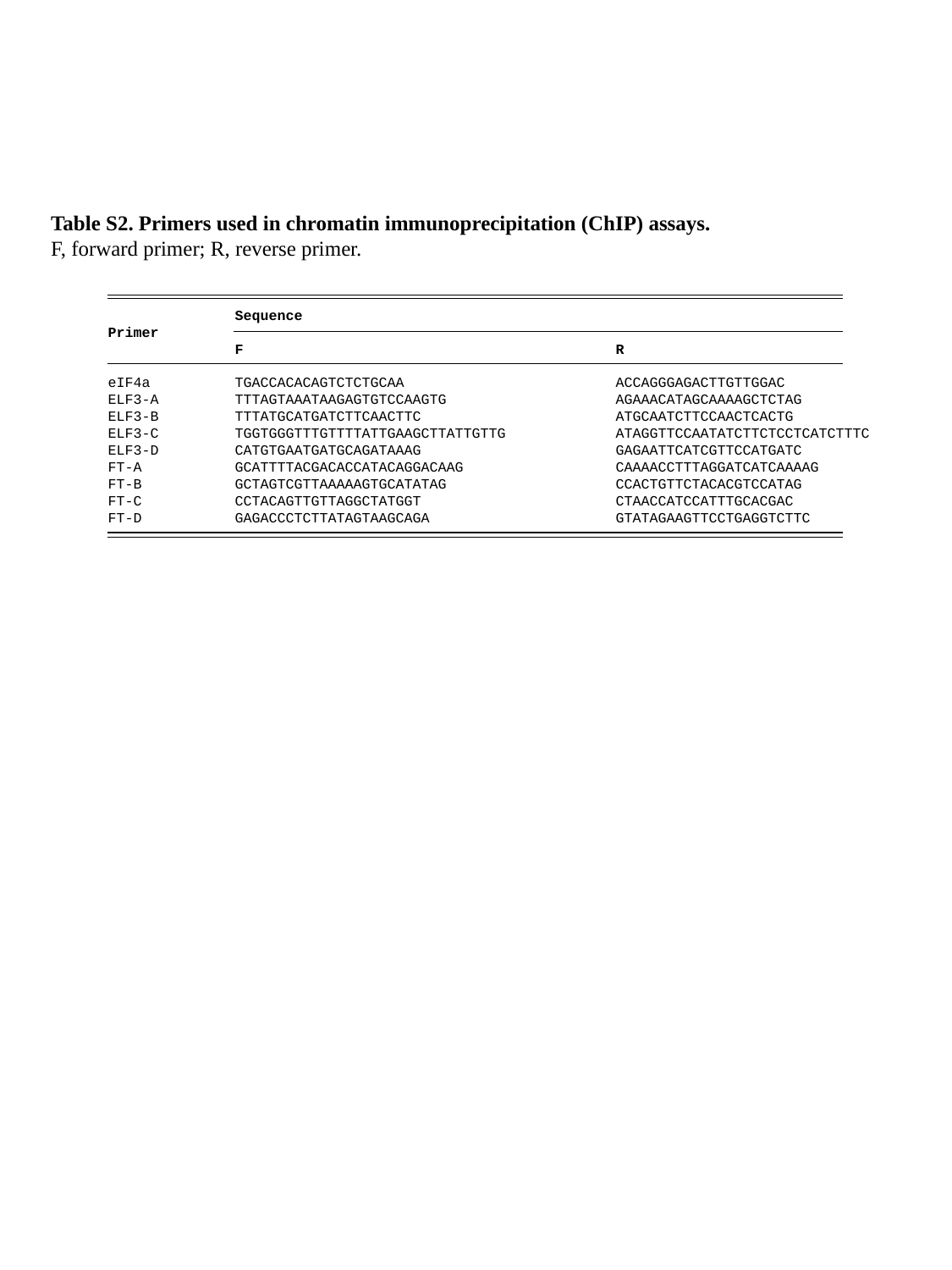

Table S2. Primers used in chromatin immunoprecipitation (ChIP) assays.
F, forward primer; R, reverse primer.
	Sequence
Primer
	F	 	R
eIF4a 	TGACCACACAGTCTCTGCAA 		ACCAGGGAGACTTGTTGGAC
ELF3-A 	TTTAGTAAATAAGAGTGTCCAAGTG		AGAAACATAGCAAAAGCTCTAG
ELF3-B	TTTATGCATGATCTTCAACTTC		ATGCAATCTTCCAACTCACTG
ELF3-C 	TGGTGGGTTTGTTTTATTGAAGCTTATTGTTG	ATAGGTTCCAATATCTTCTCCTCATCTTTC
ELF3-D	CATGTGAATGATGCAGATAAAG		GAGAATTCATCGTTCCATGATC
FT-A 	GCATTTTACGACACCATACAGGACAAG 		CAAAACCTTTAGGATCATCAAAAG
FT-B	GCTAGTCGTTAAAAAGTGCATATAG		CCACTGTTCTACACGTCCATAG
FT-C 	CCTACAGTTGTTAGGCTATGGT 		CTAACCATCCATTTGCACGAC
FT-D	GAGACCCTCTTATAGTAAGCAGA 		GTATAGAAGTTCCTGAGGTCTTC
